# Treatment Outcome of Tuberculosis Patients at Enfraz Health Center, Northwest Ethiopia: A Five-Year Retrospective Study

**DOI:** 10.1155/2014/726193

**Published:** 2014-05-05

**Authors:** Mengistu Endris, Feleke Moges, Yeshambel Belyhun, Eleni Woldehana, Ahmed Esmael, Chandrashekhar Unakal

**Affiliations:** ^1^School of Biomedical and Laboratory Sciences, University of Gondar, P.O. Box 196, Gondar, Ethiopia; ^2^Institute of Virology, Faculty of Medicine, University of Leipzig, Leipzig, Germany; ^3^Enfranz Health Center, Enfranz, Ethiopia; ^4^Department of Microbiology, Immunology & Parasitology, Debre Markos University, Debre Marqos, Ethiopia

## Abstract

*Objectives.* The aim of this study was to assess treatment outcome and associated risk factors among TB patients registered for anti-TB treatment at Enfraz health center, northwest Ethiopia. *Methods*. A five-year retrospective data (2007–2011) of tuberculosis patients (*n* = 417) registered for anti-TB treatment at Enfraz health center, northwest Ethiopia, were reviewed. Tuberculosis outcomes were following the WHO guidelines. Data were entered and analyzed using SPSS version 20. *Results*. Among 417 study participants, 95 (22.8%), 141 (33.8%), and 181 (43.4%) were smear-positive, smear-negative, and extrapulmonary tuberculosis patients, respectively. Of the 417 study participants, 206 (49.4%) were tested for HIV. The TB-HIV coinfection was 24/206 (11.7%). Seventeen study participants (4.2%) were transferred to other health facilities. Among the 400 study participants, 379 (94.8%) had successful treatment outcome (302 treatment completed and 77 cured). The overall death, default, and failure rates were 3.4%, 0.5%, and 1.2%, respectively. There was no significant association between sex, age, residence, type of TB, HIV status, and successful TB treatment outcome. *Conclusion*. Treatment outcome of patients who attended their anti-TB treatment at Enfraz health center was successful. Therefore, this treatment success rate should be maintained and strengthened to achieve the millennium development goal.

## 1. Background

Tuberculosis (TB) is a major global health problem. It causes illness among millions of people each year and ranks as the second leading cause of death from an infectious disease worldwide, next to HIV infection. According to the World Health Organization (WHO) 2012 report, almost 9 million new cases and 1.4 million TB deaths occur worldwide by the year 2011 [1]. These deaths occurred in the presence of treatments that were able to cure most of the cases.

Geographically, the burden of TB is the highest in Asia and Africa. The African region has 24% of the world's cases and the highest rates of cases and deaths per capita [1]. Almost 80% of TB cases among people living with HIV reside in Africa [1]. Among 22 high burden countries (HBCs) of TB, Ethiopia ranks the 7th with an estimated prevalence of 394/100,000 population [1].

According to the Ethiopian Ministry of Health report, TB is the leading cause of morbidity, the third leading cause of hospital admission, and the second cause of death in Ethiopia [2]. Recording and reporting the number of cases detected by national TB control programmes (NTPs) and the outcomes of treatment are of the five component packages of the directly observed therapy short-course strategy (DOTS) [1]. In 1992 Ethiopia has started the implementation of DOTS within the standardized TB prevention and control program [2]. Currently DOTS coverage is estimated at 100% geographical and 95% health facility level [1].

Ethiopia is one of the seven HBCs that reported lower rates of treatment success rate (83%) [1]. The treatment outcome monitoring in order to evaluate the effectiveness of the DOTS program is essential [3]. Furthermore, understanding the specific reasons for unsuccessful outcomes is important in order to improve treatment strategy [4]. In this regard, studies in Ethiopia ranged from 26% to 93.7% [5–13]. Previous report of the TB defaulter, failure, and death rates in Ethiopia ranged from 0.6% to 18.3% [5–8, 12–15], 0.2% to 18.6% [5–8, 12], and 2.6% to 10.1% [5–7, 9, 12, 13, 16, 17], respectively. Some of the identified independent risk factors for poor treatment outcome were being on retreatment, age being more than 55 years, being male, distance from home to treatment centre, and the added burden of using public transport to get to a treatment centre.

Despite high DOTS coverage in northwest Ethiopia, the treatment outcome of TB patients in health centers has not been assessed so far. There is little information about factors responsible for unsuccessful treatment outcome in the region. In this study, we assessed the treatment outcomes of TB patients in DOTS program and associated risk factors with unsuccessful TB treatment outcome in Enfraz health center, northwest Ethiopia.

## 2. Methods

### 2.1. Study Area

Enfraz health center is located in the northwest part of Ethiopia. Enfraz is a historic town 678 Kilometers away from Addis Ababa, capital city of Ethiopia. The health center is the only facility that renders DOTS service for the people living in and around Enfraz. Patients were diagnosed, registered, treated, and referred to other DOTS clinics following the National Tuberculosis and Leprosy Control Program (NTLCP) guideline [2].

### 2.2. Study Design and Data Collection

A retrospective study was conducted among TB patients registered from 2007 to 2011 at DOTS clinic. The sociodemographic data (such as sex, age, and residence), category of TB at the start, and the treatment outcomes of the TB patients were collected from the DOTS registration book.

### 2.3. Definitions

The Ethiopian NTLCP guideline [2], adopted from WHO, was used for the following clinical case and treatment outcome definitions.

#### 2.3.1. Smear-Positive Pulmonary TB Case

Smear-positive pulmonary TB is the case of a patient with one or more initial sputum smear examinations (direct smear microscopy) AFB-positive or one sputum examination AFB-positive plus radiographic abnormalities consistent with active pulmonary TB as determined by a clinician.

#### 2.3.2. Smear-Negative Pulmonary TB Case

Smear-negative pulmonary TB is the case of a patient with pulmonary TB who does not meet the above criteria for smear-positive disease. Diagnostic criteria should include the following: at least two AFB-negative sputum smear examinations; radiographic abnormalities consistent with active pulmonary TB; no response to a course of broad-spectrum antibiotics (except in a patient for whom there is laboratory confirmation or strong clinical evidence of HIV infection); and a decision by a clinician to treat the patient with a full course of anti-TB chemotherapy. A patient with positive culture but AFB-negative sputum examinations is also a smear-negative case of pulmonary TB.

#### 2.3.3. Extrapulmonary TB Case

Extrapulmonary TB is the case of a patient with TB of organs other than the lungs (e.g., pleura, lymph nodes, abdomen, genitourinary tract, skin, joints and bones, and meninges). Diagnosis should be based on one culture-positive specimen or histological or strong clinical evidence consistent with active extrapulmonary disease, followed by a decision by a clinician to treat a patient with a full course of anti-TB chemotherapy. A patient in whom both pulmonary and extrapulmonary TB has been diagnosed should be classified as a pulmonary case.

#### 2.3.4. Treatment Outcome

*Cured*: a patient who was initially sputum smear-positive and who was sputum smear-negative in the last month of treatment and on at least one previous occasion.* Completed treatment*: when a patient completed treatment but did not meet the criteria for cure or failure. This definition applies to sputum smear-positive and sputum smear-negative patients with pulmonary TB and to patients with extrapulmonary disease.* Died*: when a patient died from any cause during treatment.* Failed*: when a patient was initially sputum smear-positive and when a patient remained sputum smear-positive at month 5 or later during treatment.* Defaulted*: when a patient whose treatment was interrupted for 2 consecutive months or more.* Successfully treated*: when a patient was cured or completed treatment.

### 2.4. Statistical Analysis

Data were entered, cleaned, and analyzed using SPSS version 20 statistical software. Data were entered into a computer and analyzed twice by two independent investigators to ensure their quality. Bivariate and multivariate analysis were done to analyze whether there is association between the TB treatment outcomes and independent variables. Crude and adjusted odd ratio were computed and used to see the strength of association. *P* values less than 0.05 were considered statistically significant.

### 2.5. Ethical Considerations

Institutional ethical clearance was obtained from the college of Medicine and Health Sciences research ethical committee of University of Gondar.

## 3. Results

### 3.1. Characteristics of Study Participants

A total of 417 TB patients whose age ranged from 0.17 to 80 years, mean and standard deviation of 31.91 ± 1.69 years, were included in the study. Among 417 study participants 108 (52.3%) were females and 199 (47.7%) were males. Children ≤ 14 years accounted for 14.6% of the study participants. Majority of the patients were rural residents (270 (64.7%)) (Table 1).

Most of the TB patients 407/417 (97.6%) were new cases. At the start of TB treatment, the TB patients relapse, defaulter, and failure rates were 6 (1.4%), 3 (0.7%), and 1 (0.2%), respectively. The smear-positive, smear-negative, and extrapulmonary TB cases were 95 (22.8%), 141 (33.8%), and 181 (43.4%), respectively (Table 1).

Of 417 TB patients registered for anti-TB treatment 49.4% (*n* = 206) were tested for HIV. The TB-HIV co-infection was 11.7% (24/206). The smear positive, smear negative pulmonary TB and EPTB type of TB among TB-HIV co-infected patients was 41.7% (*n* = 10), 37.5% (*n* = 9), and 20.8% (*n* = 5), respectively (Table 1).

### 3.2. Treatment Outcome

The treatment outcome of most study participants was successful (302 completed their anti-TB treatment and 77 cured). The treatment failure, defaulter, and death rates were 0.5%, 1.2%, and 3.4%, respectively (Table 2).

Since the status of 17 TB patients (transferred out cases) is not known we excluded them from the treatment success rate analysis. The overall five-year treatment success rate of the TB patients was 94.8% (379/400).

The provider initiated HIV counseling and testing (PHICT) was not started in the health center up to 2008. Among the 50 in 2009, 78 in 2010, and 78 in 2011 TB patients tested for HIV, 5/50 (11.1%), 8/78 (11.4%), and 11/78 (16.4%) were positive for HIV, respectively.

In the bivariate analysis, age between 35 and 44 years and  ≥65 years showed statistically significant association with unsuccessful treatment outcome as compared to patients aged between 15 and 24 years. The multiple logistic regression showed no significant association between unsuccessful treatment outcome and the different characteristics of the TB patients including age, sex, residence, and HIV status (*P* > 0.05 in all cases) (Table 3).

In this study, in the first three years (2007–2009) smear-positive pulmonary TB was the leading type of TB (ranged from 17.9% to 27.4%), while the last two years showed an increment in smear-negative pulmonary TB cases (ranged from 20.6% to 25.5%). The highest smear-positive rate recorded was 26/99 (27.4%) in the year 2009 (Table 4). The treatment success rate of TB patients showed increment from 2007 to 2009 with 88.7% and 100% rates, respectively. In the last two years of this study (2010 and 2010) the treatment success rate was 93.4% and 94.0%, respectively (Table 4).

## 4. Discussion

Among 417 TB patients registered in DOTS clinic of Enfraz health center only 17 (4.1%) were transferred to other health institutions. Almost one out of five patients (22.8%; 95/417) registered for anti-TB treatment in the health center had smear-positive pulmonary tuberculosis. Majority of the smear-positive pulmonary tuberculosis patients (81.1%; 77/95) were cured at the end of their anti-TB treatment. This smear-positive pulmonary cure rate was comparable with reports from Tigray region (85.5%) [8].

The majority of TB patients, 379/400 (94.8%), had successful treatment outcome (cured + treatment completed). Similar successful treatment outcomes were reported by Muñoz-Sellart et al. [9] from different health institutions, Leku health center and Yirgalem health center, 90.5% and 93.7%, respectively. The present study successful treatment outcome was higher than previous reports from different parts of Ethiopia, namely, Gondar University Hospital (29.5%) [5], Felege Hiwot Referral Hospital (26%) [12], Hadiya Treatment Centers (49%) [7], Gambo Rural Hospital (66.9%) [6], Kolladiba Health Center (85.6%) [13], and Mazandaran in Iran (87.8%). The present study showed successful treatment outcome (94.8%) which met the target (90%) set in the Global Plan to Stop TB by 2015 [1].

In the present study, the prevalence of HIV among TB patients was 11.7%. This was comparable from different parts of Ethiopia such as Nekemte 11.5% [11], West Arsi 13.6% [6], and Dembia with 10.9% [13]. However, the present study TB-HIV coinfection was lower than previous reports from different health centers at Addis Ababa, Gondar University Hospital, and Felege Hiwot Referral Hospital, 27.2% [18], 52.1% [19], and 25% [12], respectively. TB-HIV coinfection in present study (11.7%) was also lower than in the WHO estimate (39%) in Africa [1]. The lowered TB-HIV coinfection in this study might be due to the higher number of rural study participants than urban residents (64.7% versus 35.5%) (Table 1).

Fourteen (3.4%) study participants died during their course of their treatment. This report was consistent with reports from Kolladiba Health Center (3.3%) [13] and different health centers in Addis Ababa (3.7%) [16] and Tigray (3.9%) [8]. The death rate of this study was lower than reports from Gondar University Hospital [5], Felege Hiwot Referral Hospital [12], and Gambo Rural Hospital [6], 10.1%, 5.8%, and 5.3%, respectively. The lower death rates in the present study might be due to the difference in the study sites, by which the study was done in rural health center, while the higher death rates in hospitals might be due to hospitalization of more critical TB patients in hospitals than in health centers. Health professionals working in DOTS clinic of the health centers might reach and advise TB patients who discontinue their anti-TB treatment than hospital workers due to their smaller catchment area and less work load. This work in turn might lead to a decrease in death rate. The other reason for low death rate in the present study might be due to the lower TB-HIV co-infection (11.7%) as compared to previous studies done at Felege Hiwot Referral Hospital (25%) [12] and Gambo Rural Hospital (13.6%) [6].

The TB treatment failure rate of this study was 0.5%, which was lower than the average TB treatment failure rate of the 22 HBCs [20]. Failure rates that ranged from 0.2% to 18.6% were reported from the different parts of Ethiopia [5–8, 12].

In this study, in the first three years (2007–2009) smear-positive pulmonary TB was the leading type of TB (ranged from 17.9% to 27.4%), while the last two years showed an increment in smear-negative pulmonary TB cases (ranged from 20.6% to 25.5%). This might be due to the increase in the HIV prevalence in the health center from year to year (11.1% in 2009 to 16.4% in 2011).

There was no significant association between unsuccessful treatment outcome and the different characteristics of the TB patients in the present study. Similar finding was reported by Datiko and Lindtjørn [17] in Southern part of Ethiopia. This result might be due to the poor characteristics of the retrospective study design to identify possible risk factors.

## 5. Conclusions

This study shows that treatment outcome of patients that attended their anti-TB treatment at Enfraz health center was successful. Enfraz health center has met the target success rate set by WHO. There was no associated risk factor identified for unsuccessful treatment outcome. Therefore, this treatment success rate should be maintained and strengthened to achieve the development goal. Further studies are needed to identify possible risk factors for unsuccessful treatment outcome.

## Figures and Tables

**Table 1:** Characteristics of TB patients with types of TB at DOTS clinic of Enfraz health center, 2007–2011.

Characteristics	Total	Type of TB		
		Smear-positive	Smear-negative	EPTB
Sex				
Male	199 (47.7)*	41 (20.6)	74 (37.2)	84 (42.2)
Female	218 (52.3)	54 (24.8)	67 (30.7)	97 (44.5)
Residence				
Urban	147 (35.3)	33 (22.4)	55 (37.4)	59 (40.1)
Rural	270 (64.7)	62 (23.0)	86 (31.9)	122 (45.2)
Age (years)				
≤14	61 (14.6)	3 (4.9)	29 (47.5)	29 (47.5)
15–24	78 (18.7)	19 (24.4)	18 (23.1)	41 (52.6)
25–34	112 (26.9)	34 (30.4)	25 (22.3)	53 (47.3)
35–44	70 (16.8)	19 (27.1)	31 (44.3)	20 (28.6)
45–54	45 (10.8)	14 (31.1)	17 (37.8)	14 (31.1)
55–64	30 (7.2)	3 (10.0)	14 (46.7)	13 (43.3)
≥65	21 (5.0)	3 (14.3)	7 (33.3)	11 (52.4)
HIV test				
Negative	182 (43.6)	39 (21.4)	65 (35.7)	78 (42.9)
Positive	24 (5.8)	10 (41.7)	9 (37.5)	5 (20.8)
Unknown	200 (48.0)	45 (22.5)	62 (31.0)	93 (46.5)
Refused to test	11 (2.6)	1 (9.1)	5 (45.5)	5 (45.5)

Total	417 (100)	95 (22.8)	141 (33.8)	181 (43.4)

*Figures in parenthesis indicate percentage, EPTB: extrapulmonary tuberculosis.

**Table 2:** Treatment outcomes of TB patients with their characteristics at DOTS clinic of Enfarz health center, 2007–2011.

Characteristics	Treatment outcomes					
	Cured	Treatment completed	Defaulter	Failure	Dead	Transferred out
Sex						
Male	32 (16.1)*	145 (72.9)	2 (1.0)	2 (1.0)	9 (4.5)	9 (4.5)
Female	45 (20.6)	157 (72.0)	3 (1.4)	0 (0.0)	5 (2.3)	8 ( 3.7)
Residence						
Urban	23 (15.6)	105 (71.4)	3 (2.0)	0 (0.0)	7 (4.8)	9 (6.1)
Rural	54 (20.0)	197 (73.0)	2 (0.7)	2 (0.7)	7 (2.6)	8 (3.0)
Age (years)						
0–14	2 (3.3)	55 (90.2)	0 (0.0)	0 (0.0)	0 (0.0)	4 (6.6)
15–24	17 (21.8)	57 (73.1)	0 (0.0)	0 (0.0)	1 (1.3)	3 (3.8)
25–34	27 (24.1)	74 (66.1)	1 (0.9)	1 (0.9)	5 (4.5)	4 (3.6)
35–44	13 (18.6)	47 (67.1)	2 (2.9)	1 (1.4)	4 (5.7)	3 (4.3)
45–54	12 (26.7)	31 (68.9)	0 (0.0)	0 (0.0)	1 (2.2)	1 (2.2)
55–64	3 (10.0)	24 (80.0)	2 (6.7)	0 (0.0)	1 (3.3)	0 (0.0)
≥65	3 (14.3)	14 (66.7)	0 (0.0)	0 (0.0)	2 (9.5)	2 (9.5)
TB type						
Smear-positive	77 (81.1)	13 (13.7)	1 (1.1)	1 (1.1)	2 (2.1)	1 (1.1)
Smear-negative	—	128 (90.8)	2 (1.4)	0 (0.0)	8 (5.7)	3 (2.1)
EPTB	—	161 (89.0)	2 (1.1)	1 (0.6)	4 (2.2)	13 (7.2)
HIV status						
Negative	35 (19.2)	133 (73.1)	1 (0.5)	0 (0.0)	7 (3.8)	6 (3.3)
Positive	7 (29.2)	13 (54.2)	1 (4.2)	0 (0.0)	1 (4.2)	2 (8.3)
Unknown	35 (17.5)	145 (72.5)	3 (1.5)	2 (1.0)	6 (3.0)	9 (4.5)
Refused to test	0 (0.0)	11 (100.0)	0 (0.0)	0 (0.0)	0 (0.0)	0 (0.0)

*Figures in parenthesis indicate percentage.

**Table 3:** Treatment success rate among TB patients at DOTS clinic of Enfraz health center 2007–2011.

Characteristics	Treatment success		OR (95% CI)	*P* value	AOR (95 % CI)	*P* value
	Yes	No				
Sex						
Male	177 (93.2)*	13 (6.8)	1.00	—	1.00	—
Female	202 (96.2)	8 (3.8)	1.85 (0.70–5.02)	0.26	0.61 (0.23–1.57)	0.30
Residence						
Urban	128 (92.8)	10 (7.2)	1.00	—	1.00	—
Rural	251 (95.8)	11 (4.2)	1.78 (0.68–4.66)	0.28	0.51 (0.19–1.30)	0.16
Age (years)						
≤14	57 (100)	0 (0.0)	0.00 (0.00–23.10)	1.00	0.00 (0.00– —)	0.99
15–24	74 (98.7)	1 (1.3)	1.00	—	1.00	—
25–34	101 (93.5)	7 (6.5)	0.19 (0.01–1.63)	0.09	5.42 (0.63–46.56)	0.12
35–44	60 (89.6)	7 (10.4)	0.12 (0.01–0.98)	0.03	7.79 (0.89–68.25)	0.06
45–54	43 (97.7)	1 (2.3)	0.58 (0.02–21.91)	0.60	1.52 (0.08–26.20)	0.77
55–64	27 (90.0)	3 (10.0)	0.12 (0.00–1.41)	0.07	7.12 (0.68–73.71)	0.10
≥65	17 (89.5)	2 (10.5)	0.11 (0.00–1.76)	0.04	6.72 (0.56–80.83)	0.13
TB type						
Smear PTB	90 (95.7)	4 (4.3)	1.00	—	1.00	—
Smear NTB	128 (92.8)	10 (7.2)	0.57 (0.15–2.06)	0.51	2.05 (0.59–7.15)	0.26
EPTB	161 (95.8)	7 (4.2)	1.02 (0.24–4.02)	0.60	1.31 (0.34–4.90)	0.69
HIV status						
Negative	168 (95.5)	8 (4.5)	1.00	—	1.00	—
Positive	20 (90.9)	2 (9.1)	0.48 (0.08–3.50)	0.31	1.06 (0.18–6. 09)	0.95
Unknown	180 (94.2)	11 (5.8)	0.78 (0.28–2.15)	0.77	1.13 (0.42–3.05)	0.81
Refused to test	11 (100.0)	0 (0.0)	Undefined	0.61	0.00 (0.00– —)	0.99

Total	379 (94.8)	21 (5.2)				

*Figures in parenthesis indicate percentage.

**Table 4:** Trends of TB type and treatment outcomes of TB patients at Enfraz heath center 2007–2011.

Treatment outcomes and TB type	Year					Total
	2007	2008	2009	2010	2011	
Category at start						
New	62 (15.2)*	88 (21.6)	98 (24.1)	77 (18.9)	82 (20.1)	407 (97.6)
Relapse	1 (16.7)	1 (16.7)	1 (16.7)	2 (33.3)	1 (16.7)	6 (1.4)
Defaulter	0 (0.0)	1 (33.3)	0 (0.0)	0 (0.0)	2 (66.7)	3 (0.7)
Failure	0 (0.0)	0 (0.0)	0 (0.0)	0 (0.0)	1 (100.0)	1 (0.2)
Tuberculosis type						
Smear-positive PTB	17 (17.9)	20 (21.1)	26 (27.4)	16 (16.8)	16 (16.8)	95 (22.8)
Smear-negative PTB	20 (14.2)	30 (21.3)	26 (18.4)	29 (20.6)	36 (25.5)	141 (33.8)
EPTB	26 (14.4)	40 (22.1)	47 (26.0)	34 (18.8)	34 (18.8)	181 (43.4)
Treatment outcomes						
Treatment success	55 (88.7)	80 (95.2)	94 (100.0)	71 (93.4)	79 (94.0)	379 (94.8)
Dead	3 (4.8)	3 (3.3)	0 (0.0)	4 (5.1)	4 (4.7)	14 (3.4)
Failure	2 (3.2)	0 (0.0)	0 (0.0)	0 (0.0)	0 (0.0)	2 (0.5)
Default	2 (3.2)	1 (1.1)	0 (0.0)	1 (1.3)	1 (1.2)	5 (1.2)
Transferred out	1 (1.6)	6 (6.7)	5 (5.1)	3 (3.8)	2 (2.3)	17 (4.1)

Overall	63 (15.1)	90 (21.6)	99 (23.7)	79 (18.9)	86 (20.6)	417 (100.0)

*Figures in parenthesis indicate percentage.
